# The Initial Attitude Estimation of an Electromagnetic Projectile in the High-Temperature Flow Field Based on Mask R-CNN and the Multi-Constraints Genetic Algorithm

**DOI:** 10.3390/s25123608

**Published:** 2025-06-08

**Authors:** Jinlong Chen, Miao Yu, Yongcai Guo, Chao Gao

**Affiliations:** Key Laboratory of Optoelectronic Technology and System, Ministry of Education, College of Optoelectronic Engineering, Chongqing University, Chongqing 400044, China; 20200801035g@cqu.edu.cn (J.C.); yumiao@cqu.edu.cn (M.Y.); gaoc@cqu.edu.cn (C.G.)

**Keywords:** electromagnetic projectile, attitude estimation, occlusion, Hamming distance constraints, genetic algorithm

## Abstract

During the launching process of electromagnetic projectiles, radiated noise, smoke, and debris will interfere with the line of sight and affect the accuracy of initial attitude estimation. To address this issue, an enhanced method that integrates Mask R-CNN and a multi-constraint genetic algorithm was proposed. First, Mask R-CNN was utilized to perform pixel-level edge segmentation of the original image, followed by the Canny algorithm to extract the edge image. This edge image was then processed using the line segment detector (LSD) algorithm to identify the main structural components, characterized by line segments. An enhanced genetic algorithm was employed to restore the occluded edge image. A fitness function, constructed with Hamming distance (HD) constraints alongside initial parameter constraints defined by centroid displacement, was applied to boost convergence speed and avoid local optimization. The optimized search strategy minimized the HD constraint between the repaired stereo images to obtain accurate attitude output. An electromagnetic simulation device was utilized for the experiment. The proposed method was 13 times faster than the Structural Similarity Index (SSIM) method. In a single launch, the target with 70% occlusion was successfully recovered, achieving average deviations of 0.76°, 0.72°, and 0.44° in pitch, roll, and yaw angles, respectively.

## 1. Introduction

In the initial phase of an electromagnetic railgun launch, accurate measurement of the projectile’s attitude is critical to mitigate initial disturbances and enhance the robustness of flight control [1]. Studies show that an electromagnetic projectile traveling at 500 m/s generates a high-temperature flow field characterized by transient temperature fluctuations exceeding several thousand Kelvins [2,3,4]. This high-temperature flow field emits intense radiation, significantly reducing the visibility of textureless metal projectiles against a bright background while also generating considerable radiation noise and a low signal-to-noise ratio (SNR). Meanwhile, the high-temperature flow field produces fragments and combustion clouds, which may occlude the electromagnetic projectile [3]. Therefore, it is essential to accurately measure the attitude of the projectile under these extreme conditions.

Monocular and binocular vision systems are widely used in industrial attitude measurement, particularly in challenging conditions such as high temperatures, intense light, and occlusions, owing to their high precision and non-contact capabilities [5,6]. Visual measurement techniques, based on feature detection and matching approaches, can generally be classified into four categories: template matching methods, neural network-based methods, geometry-based methods, and hybrid methods. Among these, template matching is the predominant technique employed for determining the attitude of projectiles. This approach involves creating pre-defined templates based on geometric shapes or characteristic patterns, then identifying the image region that best matches the template to estimate the attitude. For example, Chen Huamei et al. utilized stereo vision algorithms to determine the three-dimensional position of the projectile [7]. In the study by Hsiang-Yueh Lai et al., the three-dimensional position of the projectile was determined using stereo vision and the absolute difference technique [8]. However, significant fluctuations in lighting and occlusions can substantially affect the precision and reliability of template matching.

The geometry-based method quantifies attitude by identifying and correlating the geometric contours and topological attributes of an object [9]. Corner features, line features [10,11], circle features [12], and other geometric characteristics have important applications in the aerospace domain, especially under challenging conditions such as varying lighting, high image contrast, and noisy backgrounds [5]. For instance, in close-range orbital servicing and formation flying applications, Sumant Sharma et al. employed weak image gradient filtering to detect target edges in complex backgrounds. They applied the Hough transform to identify and register geometric constraints, such as parallel pairs, proximal pairs, and open polygons [11]. However, Jeremy A. Luckett conducted a comparative analysis on the use of ellipse, line, and point features for aerospace targets. The results showed that noise could cause the complete failure of point features, introduce significant errors in line features, and increase rotational errors in ellipse detection [13]. Andriyanov et al. employed a pseudo-gradient algorithm to estimate the translation, rotation, and scaling between images, achieving sub-pixel-level matching through iterative optimization [14]. Despite these advancements, applying geometry-based techniques in complex scenarios remains challenging, particularly as fitting elliptical characteristics requires sophisticated mathematical models [12].

Neural network-based approaches typically extract features and analyze the geometric relationships between image pairs to estimate the attitude [15]. These methods offer significant flexibility in handling complex scenarios. In fruit detection tasks [16,17,18], researchers employed YOLO for object segmentation and extracted regions of interest using bounding boxes. They then performed stereo matching on the key features within these regions. Similarly, Lijun Zhong identified critical features of the spacecraft as reference points, utilizing the coordinates of these reference points, along with the equivalent diameter, to construct a 3D bounding box for pose estimation [9]. Liming Han proposed a transformer-based pseudo-3D image matching method, which upgraded the 2D features of the source image to 3D features by introducing a reference image, and performed 3D matching from coarse to fine with the 2D features of the target image [19]. However, these methods often prioritize generality, which can pose challenges when applied to attitude estimation tasks with limited datasets.

In recent years, hybrid approaches often begin with edge segmentation algorithms to extract structured edges, as they are less sensitive to changes in lighting. Subsequently, depending on the specific characteristics, either handcrafted features or custom-designed feature points are used for object detection and matching [20]. For example, in underwater model reconstruction, Yaqin Zhou et al. performed multi-scale fusion on segmented targets and applied contour curve matching techniques to reconstruct 3D contour curves, enabling the calculation of the object’s critical dimensions [21]. For ship hulls characterized by weak textures, large scales, and complex shapes, Xiaojing Zhao et al. integrated chessboard patterns with a target to enhance the texture of the hull following image segmentation [22].

However, significant differences exist between the electromagnetic projectile and the above target. First, the negative SNR of the image causes the projectile to be almost entirely obscured by noise, making it more challenging to estimate the attitude using the gray values directly. Secondly, fragments and combustion clouds shield the electromagnetic projectile, with a maximum shielding area of 70%. In previous methods, images with strong noise interference are processed by combining segmentation algorithms to extract line features that are less sensitive to lighting changes [5] and filtering out outliers. Meanwhile, the consecutive frames of the electromagnetic projectile exhibit spatio-temporal correlation, which provides valuable insights for the context-driven repair algorithm [13,23]. However, directly applying this iterative optimization algorithm for image restoration may lead to overly complex solutions [24]. For instance, the fitness functions used in the optimization algorithm, such as root mean square error (RMSE), structural similarity index (SSIM), and peak signal-to-noise ratio, mainly depend on the gray values [25,26]. Therefore, in this study, the Mask R-CNN [27,28] was employed to segment the target region, which was then combined with the linear segment detector (LSD) [29] algorithm to outline the main edge contours. Subsequently, for the genetic algorithm, initial parameter constraints based on the centroid were established, the edge image was treated as a binary matrix [24], and Hamming distance (HD) constraints were formulated to accommodate binary targets more effectively.

The main contributions were as follows: (1) For images affected by strong noise, Mask R-CNN was used to segment the target region, which was then combined with the LSD algorithm to outline the main edge contour. (2) The initial parameter constraint was established, and an HD constraint suitable for binary targets was constructed, improving the convergence speed of the genetic algorithm and reducing the local optimization. (3) The influencing factors of the electromagnetic projectile attitude and the impact of the high-temperature flow field on the projectile attitude were analyzed.

## 2. Improved Attitude Measurement Method

**Figure 1:**
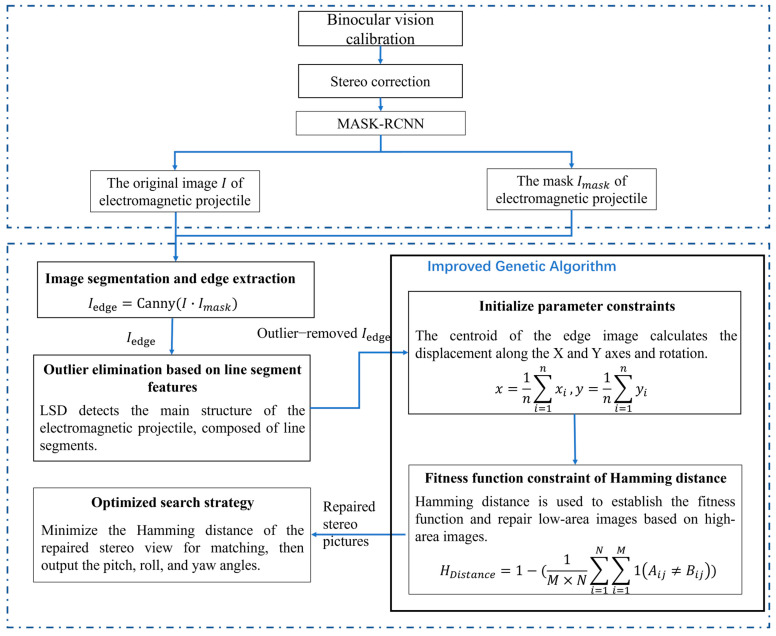
Overall structure of the proposed method.

The overall structure of the proposed method is described in Figure 1. First, the binocular images underwent stereo correction using calibration parameters to mitigate distortion and ensure horizontal alignment. Next, the images were processed through the Mask R-CNN network to extract the mask and corresponding target coordinates. The product of the mask and the original image was then fed into the Canny algorithm, resulting in the generation of an edge image. In Section 2.2, the LSD method was employed to detect lines, which helped delineate the primary structure of the target within the edge image while reducing the influence of noise. In Section 2.3, an improved genetic algorithm for image restoration was introduced. The relative displacement of the centroid of the edge image was utilized to apply initial parameter constraints. The fitness function was formulated based on HD constraints. In Section 2.4, an optimized search strategy was employed to calculate the attitude.

### 2.1. Preprocessing

The purpose of preprocessing is to accurately segment the target within the image while minimizing interference from complex backgrounds [20]. Mask R-CNN [28] is capable of generating a mask for the target. However, Mask R-CNN may face challenges in capturing fine details, especially when dealing with objects exhibiting blurred edges, inconsistent lighting, or low contrast [30]. To address this limitation, Jeba Nega Cheltha et al. introduced an adaptive Canny algorithm as a preprocessing technique for occluded objects, effectively extracting the significant edges from the input image [31]. Similarly, in complex flood scenarios, Pally R. J. et al. applied the Canny algorithm to the outputs of Mask R-CNN, Fast R-CNN, and YOLO, enabling precise detection of the water surface edges [32]. Given this, following the segmentation of the occluded electromagnetic projectile from the high-temperature flow field, the Canny algorithm [33] was employed to derive clear and well-defined edge images.

**Figure 2:**
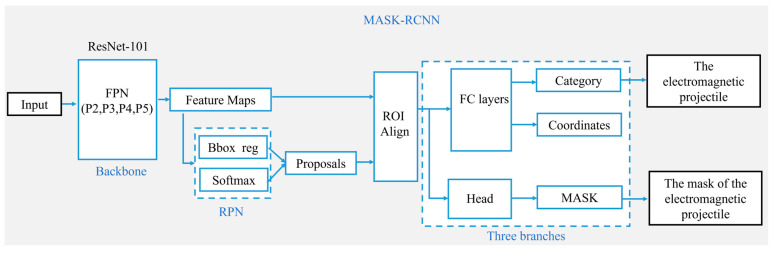
The structure of Mask R-CNN [35].

**Figure 3:**
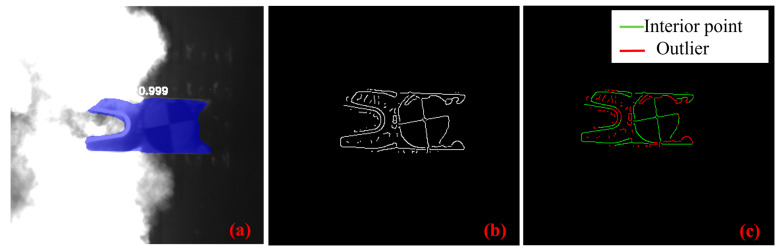
(**a**) Segmentation results obtained using Mask R-CNN. (**b**) Edge images generated from the segmented images. (**c**) Outlier removal based on line segment features.

As illustrated in Figure 2, the Mask R-CNN model formed a three-branch architecture for classification, regression, and mask prediction [34]. The ResNet-101 backbone network initially collected multi-scale feature maps from the C2 to C5 layers of the input image. Then, the feature pyramid network (FPN) created multi-scale fused feature maps from P2 to P5 by bilinearly up-sampling deep features and adding them channel-wise to shallow features. The Region Proposal Network (RPN) subsequently produced around 2000 candidate boxes by categorizing foreground anchor boxes and forecasting coordinate offsets (dx, dy, dw, and dh) to modify the anchor box locations on the P2–P5 feature maps. Non-maximum suppression (NMS) was employed to eliminate superfluous bounding boxes. The candidate boxes and feature maps were processed using the ROI Align module to extract fixed-size region features. The model concurrently produced the bounding box (coordinates), category, and high-precision binary masks for the target. The segmentation effect is shown in Figure 3a.

First, pixel-wise multiplication was performed between the original image I and the mask $I_{mask}$, effectively preserving the pixel intensities of electromagnetic projectile targets in regions with non-zero mask values. Subsequently, the Canny algorithm was applied to the masked image. These two steps can be expressed concisely by the following equation:

(1) $$I_{edge}=Canny(I\cdot I_{mask})$$

where ‘∙’ signifies the pixel-wise multiplication operation, and Canny (⋅) refers to the implementation of the Canny algorithm.

### 2.2. Outlier Removal Based on Line Segment Features

Existing studies on aeronautical target attitude estimation predominantly rely on geometric features, such as lines and circles, which are robust against substantial noise and variations in lighting conditions [5]. In contrast, electromagnetic projectiles exhibit significant differences from conventional spacecraft due to their low SNR and intense radiative light noise, which complicates the direct application of geometric features. Therefore, the LSD method was employed to detect the primary linear structure of the electromagnetic projectile from its edge image, followed by the removal of outliers [35]. The principal contour of the projectile (green region in Figure 3c) was formed by connecting short line segments. Points within 2 pixels of the principal contour were classified as inliers, whereas those further from the contour were regarded as outliers (red region in Figure 3c). By preserving the inliers and eliminating the outliers, substantial radiative noise was mitigated. The LSD parameters were configured with a line segment saliency threshold of 0.4, a gradient direction bin count of 64, an image down-sampling ratio of 0.8, and a Gaussian smoothing standard deviation of 0.6.

### 2.3. Improved Genetic Algorithm for Image Restoration

**Figure 4:**
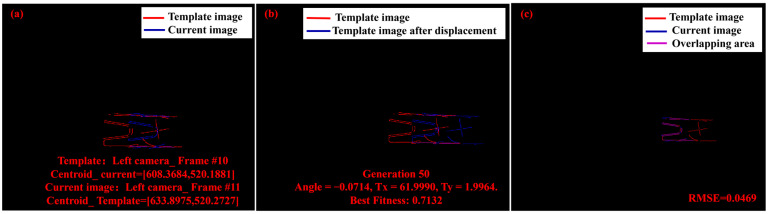
(**a**) The current image (requiring repair image) and the template image. (**b**) The template image after displacement and the original template image. (**c**) The superimposed image of the current image and the template image after displacement.

The genetic algorithm is an iterative optimization method that searches for the global optimal solution by simulating the evolutionary process [24]. In this study, the main flow of the improved genetic algorithm is shown in Algorithm 1, and the entire repair process is shown in Figure 4.

As shown in Figure 4a, the current image (requiring repair image) and the template image are displayed. In this study, with a frame rate of 10,000 fps and a velocity of approximately 500–600 m/s, the projectile stayed on the image for only about 0.0012 s during the entire filming process. The approximate displacement between consecutive attitude frames can be estimated to prevent the genetic algorithm from falling into a local optimum [35]. For adjacent frames, the movement along the x-axis was mainly influenced by the velocity, while the movement along the y-axis was primarily affected by gravity. Due to the lack of rifling in the experimental setup, the projectile experienced almost no rotation. Therefore, we constructed the initial parameter constraint according to the centroid and the quantitative offset value, which can be applied to the complete and occluded armature projectile, as shown in the following equation:

(2) $$x=\frac{1}{n}\sum_{i=1}^{n}x_{i},y=\frac{1}{n}\sum_{i=1}^{n}y_{i}$$

where *n* is the number of edge points, x and y are the coordinates of the centroid, and $x_{i}$ and $y_{i}$ are the coordinates of the edge points. In addition, there was no loss of valid points in Algorithm 1. The reason was that the target usually occupied only a small portion of the image. For example, the mask of the template image shown in Figure 4a covered 1.8% of the total image area. Furthermore, during the entire measurement process, the projectile was typically located at the center of the image, with a smaller displacement in the Y direction and a slightly larger displacement in the X direction. The image after displacement is shown in Figure 4b.

Additionally, the SSIM or RMSE similarity evaluation methods currently used in template matching algorithms have limited computational efficiency when applied to genetic algorithms. Therefore, the fitness function was defined as 1 minus HD, which served as a measure of similarity to compare the convergence process of the fitness function with SSIM. The following equation expresses the fitness function:

(3) $$Fitness\_HD=1-(\frac{1}{M\times N}\sum_{i=1}^{N}\sum_{i=1}^{M}1\left(A_{ij}\neq B_{ij}\right))$$

Here, M×N represents the size of the mask with a larger area, and $A_{ij}$ and $B_{ij}$ represent the pixel values of the two binarized images at position $\left(i,j\right)$. The function $1\left(A_{ij}\neq B_{ij}\right)$ is an indicator function that equals 1 when $A_{ij}$ and $B_{ij}$ are the same and equals 0 when $A_{ij}$ and $B_{ij}$ are different. For the two edge images, a larger Hamming distance indicated greater similarity [36], and a smaller Fitness_HD value implied better similarity.

### 2.4. Optimized Search Strategy for Attitude Calculation

The search strategy refers to the process of identifying the optimal solution or match within a specified parameter range. It is widely used in optimization problems [37] and image matching tasks [38]. Specifically, the initial displacement and rotation were approximated using the centroids of the left and right visual images. The fitness value of each individual was calculated based on the Hamming constraint. Finally, matching was performed by minimizing the fitness function, and the absolute attitude was output. In other words, steps 5 to 15 in Algorithm 1 were employed to calculate the relative displacement of the reconstructed image, while the evolutionary process outlined in steps 11 to 14 was omitted.
**Algorithm 1:** Improved Genetic Algorithm**Input:** population_size = 100; max_generations = 50; mutation_rate = 0.1; crossover_rate = 0.8; trans_x_offset = 30; trans_y_offset = 10; angle = 0; angle_offset = 5. **Output**: The best transformation parameters.1. If The area of the current image>0.4, 2. Template = select the most recent image with area greater than 0.8; 3. Else Template = the next frame; 4. End. 5. Based on Equation (2), calculate the relative displacement of the centroids of the current image and the template to obtain the relative displacement values: trans_x, trans_y. 6. Set initialization parameters and initialize the population based on the initial parameters: 7. trans_x_range = [trans_x − trans_x_offset, trans_x + trans_x_offset]; 8. trans_y_range = [trans_y − trans_y_offset, trans_y + trans_y_offset]; 9. angle_range = [angle − angle_offset, angle + angle_offset]; 10. **While** (the number of iterations ≤ max_generations) 11. Initialize the population. 10. For each individual, calculate the fitness according to Equation (3). 11. In each generation: 12. Tournament selection involves selecting the individual with high fitness as the parent. 13. Generate new offspring through two-point crossover and site mutation [24,39]. 14. Perform elitism to ensure the best individuals are preserved. 15. **end while** 16. output the fitness of these solutions. 

## 3. Measurement System and Target Analysis

### 3.1. Measurement System

**Figure 5:**
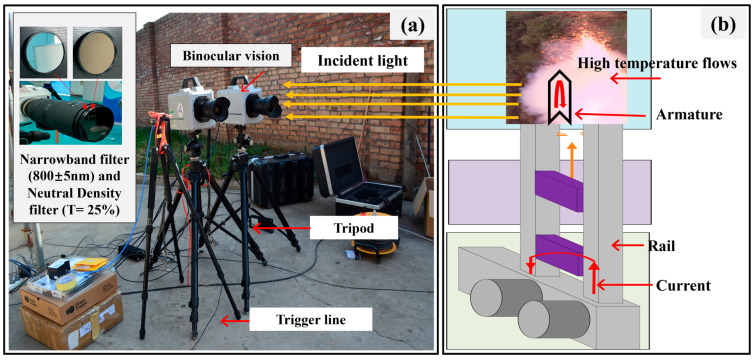
(**a**) Measurement system. (**b**) Electromagnetic emission simulation device.

As shown in Figure 5a, the binocular vision system employed in this experiment consisted of two high-speed cameras affixed to a tripod. Each camera featured an 8-bit monochrome sensor with a grayscale range of 0 to 255, a spectral response range of 400 nm to 900 nm, and a resolution of 1024 × 1024 pixels. The minimum exposure time was 1/1,000,000 s, with a frame rate of 10,000 fps. Both cameras utilized identical NIKKOR lenses (focal length: 80 mm, aperture: f/5.6), neutral density filters (T = 25%), and narrowband optical filters with a center wavelength of 800 ± 5 nm. The system was synchronized via a trigger line, with manual triggering in post-trigger mode.

Figure 5b shows the experimental setup at the Northwestern Institute of Mechanical and Electrical Engineering using the electromagnetic radiation simulation apparatus [40]. The voltage typically ranged from 1 to 10 kV, the current could reach 1 to 5 MA, and the fastest speed of the electromagnetic projectile could be as high as 2000–3000 m/s.

### 3.2. Target Analysis

**Figure 6:**
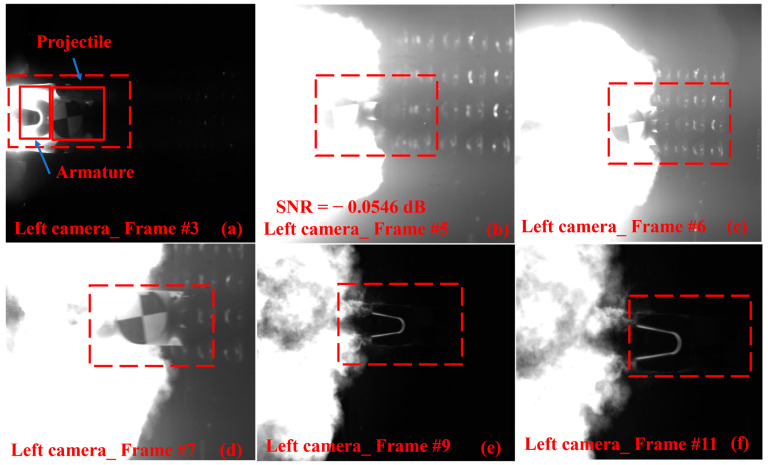
Original images captured by the left camera during a single launch operation. (**a**–**d**) Show scenarios where both the armature and a portion of the projectile were obstructed, with (**b**) representing the image exhibiting the most significant obstruction. (**e**) The unobstructed image. (**f**) Image where only the armature is visible.

To observe the dynamic changes, the electromagnetic projectile was highlighted within a red dashed box in Figure 6. The results in Figure 6a showed that the projectile consisted of both the projectile and armature. In frames 3 and 4, the projectile was just launched, leading the high-temperature flow field. Between frames 5 and 7, the flame gradually converged with the projectile, causing the most significant obstruction. In frames 8 and 9, the projectile moved ahead of the high-temperature flow field and remained unobstructed. Finally, in frames 10 to 12, only the armature was visible. Additionally, the quantization results in Figure 6b showed an occlusion area of nearly 70%, with an SNR of −0.0546 dB.

## 4. Results and Discussion

### 4.1. Experimental Calibration

In this study, binocular vision calibration was performed using the Zhang Zhengyou calibration method [34]. The projection model is given by Equation (4):

(4) $$Z_{c}\begin{array}{c} \left[\begin{array}{c} x\\ y\\ 1 \end{array}\right] \end{array}=K\left[\begin{array}{cc} R & t \end{array}\right]\left[\begin{array}{c} X_{W}\\ Y_{w}\\ Z_{w}\\ 1 \end{array}\right]$$

Here, K represents the intrinsic parameter matrix, including focal lengths and principal point coordinates. R and t are the rotation matrix and translation vector of the camera extrinsic parameters, respectively. The coordinates $X_{W},\;Y_{w},\;{and\;Z}_{w}$ are the 3D world coordinates, while x and y represent the 2D points projected onto the image plane. In other words, this method enabled the estimation of both the intrinsic and extrinsic matrices, along with the distortion model of the binocular vision system, by calculating the relationships between feature points across multiple chessboard patterns and the world coordinate system.

For calibration, GP200 calibration boards with grid squares of 15 mm were employed. The calibration board was carefully placed at the muzzle of the electromagnetic gun, while the aperture and focal length were adjusted. After ensuring the camera could capture the calibration board image accurately, 35 pairs of calibration images were collected. Calculations were performed using the MATLAB R2021a calibration toolbox. The results indicated that the average error of the binocular vision system was 0.05 pixels, which meets the required accuracy criteria. The fundamental parameters are provided in Table 1.

### 4.2. Mask R-CNN Performance Analysis

**Figure 7:**
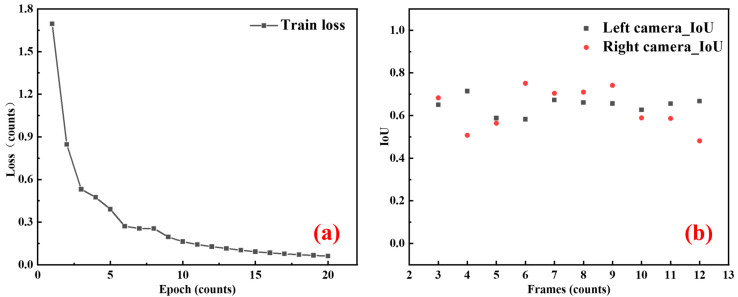
(**a**) The losses incurred during the training procedure. (**b**) The IoU curve for the recognition of electromagnetic projectiles.

The hardware configuration and hyperparameters of the Mask R-CNN model are shown in Table 2. In this experiment, a total of 50 images were used for training, while 20 images were used for testing. The model was trained for 20 iterations, each consisting of 100 gradient update batches. The loss function progress during training is shown in Figure 7a. Notably, the model exhibited significant convergence within the first 10 training cycles, with the loss value dropping sharply from 0.89 to 0.21. In subsequent iterations, the model showed a stable convergence trajectory, stabilizing at a loss value of 0.06. The model’s performance was evaluated using the Intersection over Union (IoU) [27], a key metric for image semantic segmentation tasks. IoU measures the overlap between the predicted and ground truth masks, with a value above 0.5 indicating acceptable segmentation accuracy. As shown in Figure 7b, the average IoU for target segmentation in the test set was 0.631, meeting the standards for segmenting target areas in complex backgrounds.

### 4.3. Edge Segmentation and Genetic Algorithm Performance Analysis

**Figure 8:**
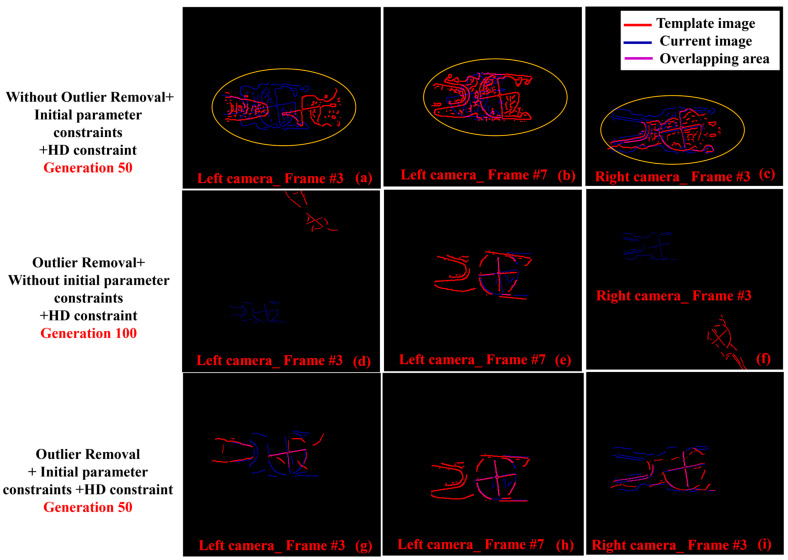
Performance evaluation of outlier removal and initial parameter constraints. (**a**–**c**) The repair effect without outlier removal, with local optimizations highlighted by yellow boxes. (**d**–**f**) The repair effect without the initial parameter constraints. (**g–i**) The repair effect when both outlier removal and the initial parameter constraints were applied.

To evaluate the effectiveness of outlier removal and initial parameter constraints, three sets of comparative experiments are presented in Figure 8. In the first row of Figure 8, when outlier removal was not applied, local optimization occurred. In the second row of Figure 8, when initial parameter constraints were not applied, local optimization was still encountered after 100 generations. The third line of Figure 8 shows that the proposed algorithm had good performance. In short, this demonstrated that outlier removal effectively reduced the likelihood of local optimization. The initial parameter constraints not only improved the convergence speed but also decreased the probability of local optimization.

**Figure 9:**
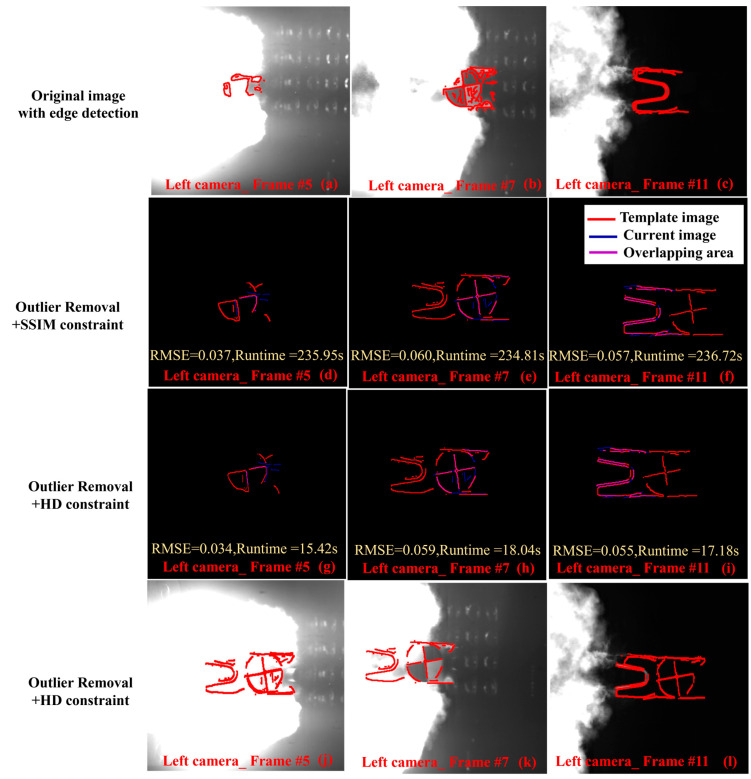
Grouped tests of edge segmentation incorporating SSIM and HD constraints. (**a**–**c**) are edge detection of the original image, (**d**–**f**) are outlier removal + SSIM constraint, (**g**–**i**) are outlier removal + HD constraint, and (**j**–**l**) are outlier removal + HD constraint overlayed on the original image, where (**j**) underwent two rounds of restoration.

To further evaluate the fitness function performance of the genetic algorithm under the SSIM and HD constraints, three representative image sets were selected for processing. As shown in the first line of Figure 9, these images were from frames 5, 7, and 11 of the left camera. The processing results are shown in the second and third lines of Figure 9. The RMSE values for both images were nearly identical, approximately 0.052. However, the convergence rate using the HD constraint was about 13 times faster than that using the SSIM constraint. Finally, as shown in the last row of Figure 9, the edge image was overlaid onto the original image to demonstrate the effectiveness of the proposed method in repairing the image. Notably, in the case shown in Figure 9a, where the occluded area reached 70%, the first repair result is shown in Figure 9g and the second in Figure 9j.

**Figure 10:**
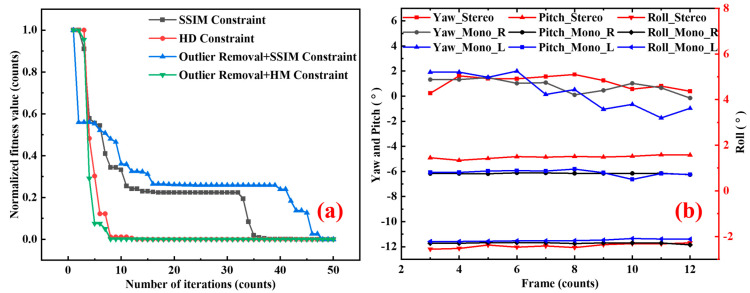
(**a**) Convergence curves for various methods. (**b**) Results of attitude measurement for the electromagnetic projectile.

The results indicate that the proposed method effectively reduced background noise interference in complex occluded scenes and could repair up to 70% of the maximum occluded target area. In addition, the convergence speed of the algorithm is shown in Figure 10a. The results showed that the removal of outliers had no significant effect on the improvement of convergence speed. In comparison, the HD constraint provided a much faster convergence rate than SSIM.

### 4.4. Attitude Measurement Performance Analysis

**Figure 11:**
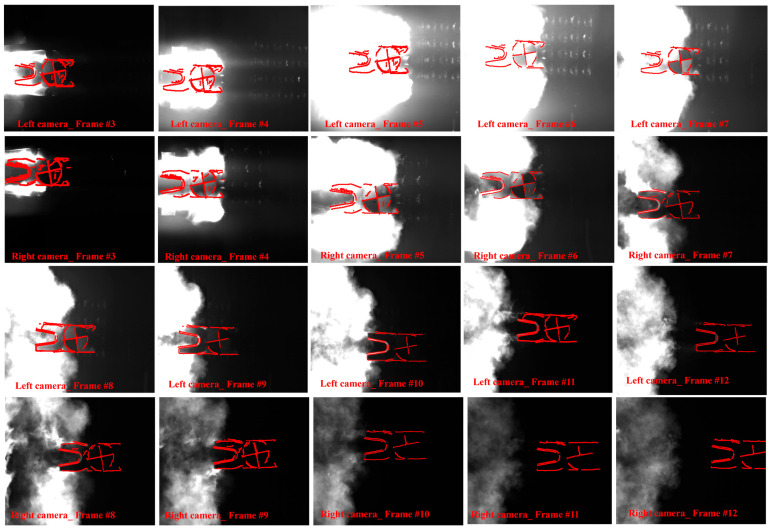
Diagram illustrating the repair effect throughout the complete initial posture measurement process of a single experiment.

As shown in Figure 11, the image was repaired using the method described in Section 2.3. Verifying the electromagnetic projectile’s attitude against ground truth was inherently challenging, with the primary sources of error stemming from lighting and occlusion. Compared to stereo vision, the variation of feature points between adjacent frames in continuous images was relatively small. Therefore, the left and right cameras of the stereo vision system were treated as independent monocular vision systems, which served as references for calculating the deviation. The rolling, pitch, and yaw angles for monocular and stereo vision were computed using the same methods outlined in Section 2.4.

The point-line graph in Figure 10b illustrates the results of the posture calculation. For the rolling angle, the outcomes from the left, right, and stereo vision systems were nearly identical, displaying constant deviations of 0.7245 and 0.6887 between the stereo vision and both the left and right vision systems. This constant deviation can be attributed to the lack of rifling in the electromagnetic experimental setup, which caused the rolling angle to remain virtually unchanged. The average pitch angle was −11.71°, with average deviations of 0.2440 and 0.4400. The significant pitch angle was likely due to inconsistencies in the mass of the projectile and armature, influenced by gravitational forces, resulting in a noticeable downward trend. This can be verified by observing the projectile’s landing point and the entire launch sequence. The yaw angle ranged from 0.23° to 1.72°, with deviations of 0.53° and 0.76°. Notably, frames 7 to 11 corresponded to the period after the projectile exited the high-temperature flow field. Based on our previous research on the high-temperature flow field [3], it was speculated that the variation in yaw angle was influenced by airflow within the high-temperature flow field during the temperature rise phase between frames 6 and 8.

## 5. Conclusions

This study presented an enhanced method that integrated Mask R-CNN with a multi-constraint genetic algorithm, aiming to accurately measure the initial posture of an electromagnetic projectile in a high-temperature flow field. Experimental results showed that the combination of Mask R-CNN and Canny edge detection effectively outlined the edges of the electromagnetic projectile. Outlier removal, based on line segment characteristics, significantly reduced the risk of local optimization. In the refined genetic algorithm, initial parameter constraints derived from the target’s centroid, along with a fitness function based on Hamming distance, resulted in a convergence rate that was 13 times faster than that of the traditional SSIM method. Ultimately, the proposed method was utilized to restore images of the entire initial process of the electromagnetic projectile, achieving a recovery rate of up to 70% of the maximum occluded target area. The calculated deviations between stereo vision and the left–right vision system were as follows: the fixed deviations for the rolling angle were 0.7245 and 0.6887, while the average deviations for the pitch angle were 0.2440 and 0.4400. The yaw angle demonstrated greater deviations, with values of 0.53 and 0.76, respectively. A detailed analysis of the posture measurement results was conducted. This method demonstrated a strong capability in accurately measuring the initial posture of the electromagnetic projectile.

## Figures and Tables

**Table 1:** Basic parameters of the binocular vision system.

Parameter	Camera Parameters	Left Camera	Right Camera
Camera intrinsics	Focal length	[3549.3, 3548.9]	[3565.8, 3565.7]
	Main point	[521.7, 189.8]	[512.3, 178.0]
	Radial distortion	[−0.04, 3.30]	[−0.004, 2.29]
	Tangential distortion	[0, 0]	[0, 0]
Camera extrinsics	Rotation matrix	$\left[\begin{array}{ccc} 0.9880 & -0.0025 & 0.1547\\ 0.0020 & 1.0000 & 0.0035\\ -0.1547 & -0.0031 & 0.9879 \end{array}\right]$	
	Translation matrix	$\left[\begin{array}{ccc} 732.2398 & -0.2567 & 34.4860 \end{array}\right]$	

**Table 2:** Hardware configuration and hyperparameters.

System Configuration and Hyperparameters	Parameter
computing platform	16 GB of RAM
	NVIDIA GeForce RTX 3060 Ti GPU
	Intel i5-12400KF CPU
input image resolution	320 × 384 pixels
network parameters	Adam optimizer
batch size	2
initial learning rate	0.001

## Data Availability

No data was used for the research described in the article.
